# Assessment of PGP traits of *Bacillus cereus* NDRMN001 and its influence on *Cajanus cajan* (L.) Millsp. phytoremediation potential on metal-polluted soil under controlled conditions

**DOI:** 10.3389/fpls.2022.1017043

**Published:** 2022-10-13

**Authors:** Mathiyazhagan Narayanan, Arivalagan Pugazhendhi, Ying Ma

**Affiliations:** ^1^ Division of Research and Innovations, Department of Biotechnology, Saveetha School of Engineering, Saveetha Institute of Medical and Technical Science, Tamil Nadu, India; ^2^ School of Engineering, Lebanese American University, Byblos, Lebanon; ^3^ College of Resources and Environment, Southwest University, Chongqing, China

**Keywords:** metal contamination, metal tolerant bacteria, PGP characteristics, *C. cajan* (L.) Millsp., greenhouse study

## Abstract

The current study looked at the plant growth-promoting (PGP) traits of the pre-isolated and metal-tolerant *Bacillus cereus* NDRMN001 as well as their stimulatory effect on the physiology, biomolecule content, and phytoremediation potential of *Cajanus cajan* (L.) Millsp. on metal-polluted soil. The bauxite mine, which is surrounded by farmland (1 km away), has been severely polluted by metals such as Cd (31.24 ± 1.68), Zn (769.57 ± 3.46), Pb (326.85 ± 3.43), Mn (2519.6 ± 5.71), and Cr (302.34 ± 1.62 mg kg^−1^) that exceeded Indian standards. The metal-tolerant *B. cereus* NDRMN001 had excellent PGP activities such as synthesis of hydrogen cyanide (HCN), siderophore, indole acetic acid (IAA), N_2_ fixation, and P solubilization. Furthermore, the optimal growth conditions (temperature of 30°C, pH 6.5, 6% glucose, 9% tryptophan, and 1.5% tricalcium phosphate) for effective synthesis and expression of PGP traits in *B. cereus* NDRMN001 were determined. Such metal-tolerant *B. cereus* NDRMN001 traits can significantly reduce metals in polluted soil, and their PGP traits significantly improve plant growth in polluted soil. Hence, this strain (*B. cereus* NDRMN001) significantly improved the growth and phytoremediation potential of *C. cajan* (L.) Millsp on metal-polluted soil without [study I: 2 kg of sieved and autoclaved metal-polluted soil seeded with bacterium-free *C. cajan* (L.) Millsp. seeds] and with [study II: 2 kg of sieved and autoclaved metal-polluted soil seeded with *B. cereus* NDRMN001-coated *C. cajan* (L.) Millsp. seeds] *B. cereus* NDRMN001 amalgamation. Fertile soil was used as control. The physiological parameters, biomolecule contents, and the phytoremediation (Cr: 7.74, Cd: 12.15, Zn: 16.72, Pb: 11.47, and Mn: 14.52 mg g^−1^) potential of *C. cajan* (L.) Millsp. were significantly effective in study II due to the metal-solubilizing and PGP traits of *B. cereus* NDRMN001. These results conclude that the test bacteria *B. cereus* NDRMN001 considerably improved the phytoremediation competence of *C. cajan* (L.) Millsp. on metal-polluted soil in a greenhouse study.

## Introduction

Soil is an important factor in many ecological processes, and majority of the creatures that live on Earth rely on it for the successful completion of their lives (Kang et al., 2018). Unfortunately, industrial revolution and modern agricultural practices have caused widespread soil pollution over the last few decades (Khan et al., 2017). Open cast mining, metalliferous industries, dyeing industries, and the frequent application of synthetic pesticides and fertilizers are major sources of soil pollution (Minari et al., 2020). Surface mining industries, for example, cause massive heavy metal pollution by dumping unprocessed waste mine tailings as a heap (Minari et al., 2020). Erosional events have dispersed the metal components from heap to the surrounding land and water reservoirs. Residents near mining sites continue to farm despite being unaware of metal pollution, which may increase the risk of toxic metals spreading to living beings through the utilization of plant-based foods originating from heavy metal-contaminated sites (Anning and Akoto, 2018). Some plants’ root systems can absorb a certain amount of metals from the soil and transfer them to their above-ground parts (Pinto et al., 2018). The most common chemical and physical treatments are not suitable for large-scale metal removal, and they may cause secondary pollution on soil; additionally, such methodologies are not really economically feasible (Allamin et al., 2020). Another popular technique is microbial bioremediation using several numbers of bacteria (*Bacillus* sp., *Pseudomonas* sp., *T. ferrooxidans*, *Actinobacteria* sp., *Staphylococcus* sp., etc.) and fungi (*Aspergillus* sp., *Penicillium* sp., *Mucor* sp., and so on) (Kumar et al., 2019) for metal remediation. Nevertheless, the majority of the processes are really only appropriate for small-scale treatment. Therefore, the phytoremediation technique is the better option for large-scale methods of soil reclamation with an eco-friendly solution, though it has some disadvantages when applied to metal-polluted soil (Gavrilescu, 2022). Only a few plant species have been identified as metal-tolerant and capable of metal remediation (Alshaal et al., 2015). Thus, individual plant-based phytoremediation and microbial remediation approaches have become less effective in metal-polluted soil because the massive heavy metal contents reduce the seed germination percentages and are toxic to the plants by first interfering with the biosynthetic pathways of chlorophyll pigments in plants (Rai et al., 2021). It causes decreased photosynthesis, which results in poor plant growth and biomass yield. The integrated approach to remediate metal-contaminated site remediation was also efficient (Hussain et al., 2019). The plant–microbe interactions play the most important role in the reclamation process. Because metal-tolerant microbes can convert toxic metals to non-toxic ones, the plant could easily absorb metals with the aid of metal-susceptible bacteria (Rane et al., 2022). Furthermore, rhizobacteria with plant growth-promoting (PGP) characteristics together with metal resistance could be a promising candidate for improving growth and phytoremediation competence in metal-polluted soil (Allamin et al., 2020). *Cajanus cajan* (L.) Millsp. has previously been identified as an effective phytoremediation agent in metal-enriched mine tailings (Mathiyazhagan and Natarajan, 2013). Furthermore, the pre-identified *Bacillus cereus* NDRMN001 was shown to be an excellent metal-tolerant and degradation bacterium (as it was isolated from the metal-polluted site) on metal-polluted soil (Ma et al., 2013). Hence, this research was framed to evaluate the PGP characteristics of *B. cereus* NDRMN001, and its optimistic influence on physiological parameters, biomolecule contents, and the phytoremediation efficiency of *C. cajan* (L.) Millsp. grown on metal-contaminated soil under greenhouse conditions was also investigated.

## Materials and methods

### Soil sample collection and handling

**Figure 1:**
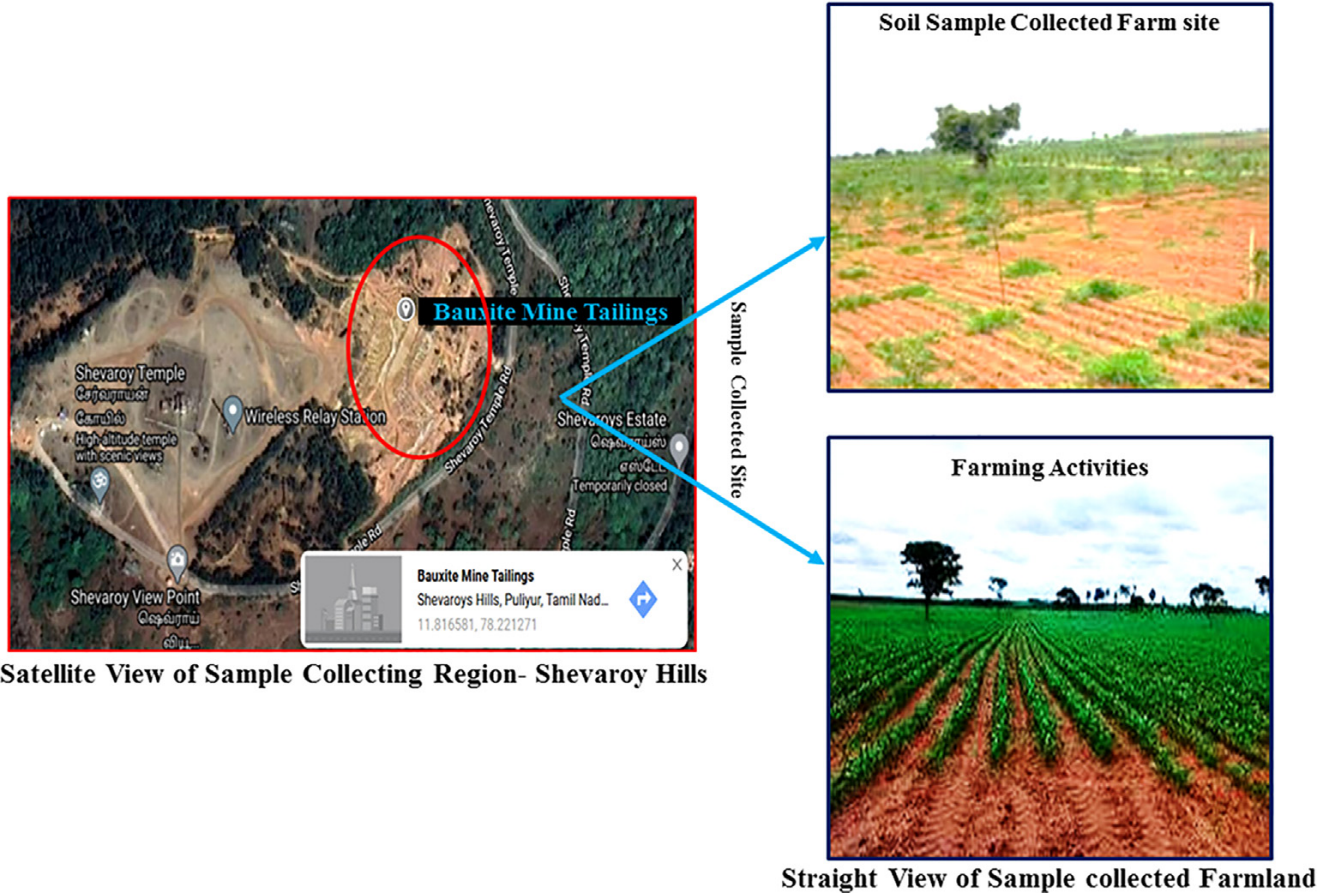
Satellite and straight view of study area. Red circle indicates heaps of abandoned bauxite mine tailing.

About 200 kg of stones and dust-free soil sample (sieving by sand filter: mesh size 140–200 and soil particle size was approximately 0.66 to 0.78 mm) was collected in ethanol-wiped sack from the agricultural land located 1 km away (11.815399, 78.222725) from the bauxite mine (11.816581, 78.221271) of Yercaud, Salem district, Tamilnadu, India (**Figure 1**). About 10 soil samples were collected at a depth of 10 cm from nearby mining areas and combined into one sample. The collected sample was immediately transferred to the laboratory for additional processes such as physicochemical property analysis, and the sample was prepared for the phytoremediation experiment under semi-controlled conditions. The fertile soil from the Sri Ram nursery garden, Salem, Tamil Nadu, India was utilized in this investigation as the control soil. Moreover, for agricultural operations, the sample collection site was initiated (**Figure 1**).

### Soil characterization

The physicochemical properties of the soil are the most important factor determining the growth and yield of plant biomass. Parameters such as pH, electric conductivity (EC; dSm^−1^), and N, P, K, Cd, Pb, Mn, Zn, Cr, Fe, and M contents of the test and fertile soil (control) samples were analyzed using Narayanan et al.’s protocol with slight modifications (Narayanan et al., 2020a). Concisely, the pH and EC of soil samples were determined using pH and conductivity meters, respectively. The essential and metal element contents of 100 mg of soil samples were investigated using the acid digestion method (digested with a 1:3 ratio of Conc. HNO_3_ and HCl) pre- and post-treatment. Whatman No. 0.5 filter paper was used to separate the elemental extracts. The extracted metals of each sample were examined through inductively coupled plasma–optical emission spectrometry (ICP-OES, Perkin-Elmer, USA).

### Brief profile of test bacterium

The pre-identified metal-tolerant *B. cereus* NDRMN001 (Narayanan et al., 2020b) was used in this study. It was isolated from an adjacent bauxite mine tailings site; it is a Gram-positive, aerobic-to-facultative, spore-forming rod; and it also possesses impressive tolerance against various metals (Cr, Pb, Zn, Mn, and Cd), up to 700 mg L^−1^ concentration of metals and their degradation potential as demonstrated by a lab-scale bioremediation investigation. This bacterial isolate was used in this research to evaluate their PGP characteristics, as well as their assistance in plant growth and the phytoremediation potential of the selected plant species *C. cajan* (L.) Millsp.

### Qualitative determination of PGP traits of *B. cereus* NDRMN001

#### Ammonia (NH_3_), hydrogen cyanide (HCN), siderophore, nitrogen (N_2_) fixing efficiency determination, phosphate-solubilizing, and indole acetic acid-producing potential analyses

The ammonia (NH_3_)-producing potential of *B. cereus* NDRMN001 was investigated using the Prakash et al. methodology with minor modifications (Prakash et al., 2019). In brief, a log phase culture of *B. cereus* NDRMN001 (absorbance = 1.1 ± 0.021) was inoculated onto 6 ml of sterilized peptone broth and incubated at 30° for 3 days. Approximately 0.5 ml of Nessler’s reagent was added to the culture-grown tubes and the color changes observed from brown to yellow were deemed positive. The standard method was used to assess *B. cereus* NDRMN001 ability to produce HCN. A loop full of log phase *B. cereus* NDRMN001 was streaked on an LB agar plate nourished with glycine (4.4 g L^−1^). A layer of Whatman No. 1 filter paper moistened with 0.5% picric acid (enriched with 2% of Na_2_CO_3_) was placed on top of the culture-grown medium. Furthermore, the plates were wrapped in parafilm and cultured at 30°C for 4 days, before the color changes from light orange into light red were noted and considered positive for HCN production by the test isolate. The siderophore synthesis efficacy of *B. cereus* NDRMN001 was investigated using the methodology of Bruno et al. (2020) with minor modifications. *B. cereus* NDRMN001 was inoculated on the Chrome Azurol S agar (selective media) and cultured at 30° for 76 h. The appearance of a yellow to orange glow around the ridges of colonies was interpreted as a sign of siderophore secretion. Furthermore, the N_2_ fixing ability of *B. cereus* NDRMN001 was investigated on Ashby medium using the Checcucci et al. protocol with some modifications (Checcucci et al., 2017). Concisely, the medium was made with glucose (2.5 g), mannitol (2.5 g), calcium carbonate (5 g), sodium molybdate dihydrate (0.001 g), calcium chloride dihydrate (0.1 g), magnesium sulfate heptahydrate (0.1 g), potassium dihydrogen phosphate (0.1 g), ferrous sulfate heptahydrate (0.01 g), dipotassium hydrogen phosphate (0.9 g), and agar-agar (16 g L^−1^) in sterilized double-distilled water. About 0.1 ml of pure *B. cereus* NDRMN001 (absorbance = 1.1 ± 0.021) was inoculated by the spread plate method on the sterilized Ashby medium-containing plate. The inoculated plates were incubated at 30° for 72 h. After incubation, the colonies with brown to black pigment were considered positive for nitrogen fixation.

Similarly, the phosphate-solubilizing and IAA-producing potential of test isolate were determined as follows. In brief, 100 µl of *B. cereus* NDRMN001 was inoculated on the sterilized Pikovskaya’s agar (PKA) medium (pH 7.1 ± 0.3) *via* the pour plate method and cultured at 30° for 48 h (Nayak et al., 2019). After incubation, well-grown cultures (colonies) with halo zones were deemed positive for phosphate solubilization. The IAA-producing potential of *B. cereus* NDRMN001 was analyzed by inoculating 100 µl of culture on LB broth supplemented with 1% tryptophan and incubation at 30° for 72 h. After incubation, the culture was spun at 10,000 *g* for 8 min, and then about 2 ml of ready-made Salkowski solution was mixed to 1 ml of pellet-free supernatant and later three drops of H_3_PO_4_ (orthophosphoric acid) was added, and then kept under dark conditions for 2 h. Later, the absorbance of this mixture was measured at 530 nm using commercial tryptophan as the standard and the result was recorded.

### Quantitative analysis of PGP traits of *B. cereus* NDRMN001

#### HCN, siderophore, nitrogen fixation, IAA secretion, and P solubilization quantitative analyses

About 2 ml of *B. cereus* NDRMN001 (absorbance = 1.1 ± 0.021) was inoculated into 100 ml of King’s B broth medium enriched with 4.4 g L^−1^ of glycine. A 10 × 0.4-cm filter paper strip was drenched in alkaline picrate solution and retained hung from the flask’s neck before being sealed with parafilm. Furthermore, these flasks were kept at 30°C in a shaker incubator at 150 rpm for 4 days. Following incubation, the color of the sodium picrate filter paper was changed to reddish corresponding to the amount of HCN produced. The color was extracted from the filter paper by placing it in a flask with 10 ml of distilled water. The absorption of the color dissolved in the distilled water was measured at 625 nm using a spectrophotometer, and the distilled water was used as a blank (Ullah et al., 2015; Subrahmanyam et al., 2018) and compared with the growth of the cell. The ability of *B. cereus* NDRMN001 to produce siderophores was evaluated using a standard CAS liquid assay with minor modifications. After blending 500 µl of the centrifuged culture supernatant with 500 µl of CAS assay, 10 μl of shuttle solution was added and the mixture was left undisturbed for a few minutes. The siderophore development was shown to decrease the blue color of the solution. The color intensity was noted at 630 nm using a spectrophotometer and was correlated with the cell’s growth. The following formula was used to calculate the percentage of siderophore:


$$\mathrm{Percentage of Siderophore Units}=\frac{\mathrm{(Absorbance of reference - Absorbance of sample)}}{\mathrm{Absorbance of reference}}\;\mathrm{x100}$$

The nitrogen-fixing potential of *B. cereus* NDRMN001 was investigated on nitrogen-free Jensen’s medium using the protocol of Checcucci et al. (2017). About 1 ml (absorbance = 1.1 ± 0.021) of *B. cereus* NDRMN001 in the logarithmic phase was added into 250 ml of Jensen’s broth medium (N_2_-free) in a 1-L glass flask. The inoculated flask was cultured at 32° for 14 days with a 60-ppm dosage of atmospheric N_2_ in a gas chamber. The high T° catalytic oxidation (HTCO) method was used to calculate the rate of nitrogen fixation in the medium by the 1st and 14th day’s nitrogen content analysis. The total volume of nitrogen fixed by *B. cereus* NDRMN001 was estimated by the following formula:


$$\mathrm{Rate of N fixation}=\frac{\mathrm{(Amt. N in cell after incubation- Amt. of N in cell at intial day incubation)}}{\mathrm{(Incubation period) X Mass of dry weight of cell}}\;$$

The IAA secreting ability of *B. cereus* NDRMN001 was investigated using the protocol established by Fahad et al. with some minor modification (Fahad et al., 2015). In brief, 0.1 ml of *B. cereus* NDRMN001 was poured into the LB broth containing 3% tryptophan in a separate flask. Each flask was incubated in an orbital shaker incubator for about 76 h with 120 rpm at 30°. Then, culture contained in each flask was spun at 10,000 rpm for 10 min. About 1 ml of culture supernatant (pellet free) was collected and 2 ml of ready-made Salkowski solution was added and then three drops of H_3_PO_4_ was added and then kept under dark conditions for 2 h. The optical density (OD) of each reaction mixture was documented at 530 nm by comparison with various dosages (20–320 µg ml^−1^) of standard IAA. The graph was plotted, and the quantity of IAA synthesis at various dosages of tryptophan was measured and correlated with cell growth. The P-solubilizing potential of *B. cereus* NDRMN001 was analyzed as per the methodology of Narayanan et al. with required changes (Narayanan et al., 2020b). In brief, about 0.1 ml of *B. cereus* NDRMN001 was added into 100 ml of freshly prepared Pikovskaya’s medium supplemented with 0.5 g of tricalcium phosphate in individual flasks and cultured for 2 weeks at 30° with 120 rpm in a shaker incubator and a separate control was maintained. Later, these were centrifuged at 8,000 rpm for about 10 min, and the soluble P in the supernatant was studied using the Mo blue technique. Moreover, soluble P absorption was compared to cell growth.

#### Optimization of growth parameters

The optimum growth conditions for IAA production, nitrogen fixation, HCN and siderophore production, and P solubilization were studied for *B. cereus* NDRMN001 using the one*-*factor-at-a-time mode (Narayanan et al., 2020b). About 100 µl of *B. cereus* NDRMN001 (absorbance = 1.1 ± 0.021) was cultured in 250 ml of nitrogen-free LB broth supplemented with various dosages of glucose (2%, 4%, 6%, 8%, and 10%), tryptophan (3%, 6%, 9%, 12%, and 15%), and tricalcium phosphate (0.5%, 1.0%, 1.5%, and 2%) at various pH values (5.5, 6.5, 7.5, and 8.5), temperatures (25, 30, 35, and 40°), and days of incubation (3, 6, 9, 12, and 15). Triplicates were performed for each growth parameter to get accurate and reproducible results. The aforementioned respective protocol was followed for each parameter quantification analysis.

#### Production of IAA, HCN, and siderophore, nitrogen fixation, and P solubilization under optimized conditions

Based on the results attained from the optimization study, the PGP traits (production of IAA, HCN, and siderophore, N_2_-fixing potential, and P solubilization) of *B. cereus* NDRMN001 were studied at 30°, pH 6.5, 6% of glucose, 9% of tryptophan, and 1.5% of tricalcium phosphate (Narayanan et al., 2020b). Concisely, 1 ml of phase B of the log phase *B. cereus* NDRMN001 (absorbance =1.1 ± 0.021) was inoculated by triplicate analysis into 250 ml of nitrogen-free LB broth medium in a 500-ml flask modified with the above parameters. Then, they were cultured for 12 days at 30°. The growth kinetics of *B. cereus* NDRMN001 (600 nm) during incubation along with IAA (530 nm) and soluble phosphate (660 nm) levels were analyzed on a 2-day interval for up to 12 days using a UV double beam spectrophotometer (Ma et al., 2015; Franchi et al., 2017).

#### Phytoremediation potential of *Cajanus cajan* (L.) Millsp. with and without the amalgamation of *B. cereus* NDRMN001 on metal-contaminated soil

According to the earlier description by Mathiyazhagan and Natarajan (2013), *C. cajan* (L.) Millsp. was chosen for this study to assess its metal reclamation potential on metal-polluted soil with and without the amalgamation of multipotential *B. cereus* NDRMN001. The experimental setup was designed according to Narayanan et al. (2020b) under greenhouse conditions. Briefly, the quality seeds of *C. cajan* (L.) Millsp. were procured. About 1:1 proportions of 30% H_2_O_2_ as well as ethanol (v/v) were used to sterilize the seed surfaces for 5 min, and the seeds were then rinsed twofold with sterile distilled water, then air dried, and stored in sterilized polythene bags. The logarithmic stage *B. cereus* NDRMN001 [10^6^ colony-forming unit (CFU) ml^−1^] was coated on the surface of the sterilized *C. cajan* (L.) Millsp. by sinking the seeds in the bacterial culture for 20 min, and the seeds were air dried in a sterile chamber. The bacterial coat-free seeds were used as the control.

The experiment was carried out in nursery grade (80-micron) polythene bags with dimensions of 125 + 100 * 225 nm (2.5 L volume). The experimental setup was framed as follows: study I: 2 kg of sieved and autoclaved metal-polluted soil seeded with bacterium-free *C. cajan* (L.) Millsp. seeds (15 No.); study II: 2 kg of sieved and autoclaved metal-polluted soil seeded with *B. cereus* NDRMN001-coated *C. cajan* (L.) Millsp. seeds (15 No.). Furthermore, 2 kg of sterilized fertile nursery garden soil seeded with bacterium-free *C. cajan* (L.) Millsp. seeds (15 No.) was used as the control. About 50 g of the autoclaved metal-free goat manure was added to all the groups as the primary fertilizer. Triplicates (excluding control) were performed for all the studies. The plantation study was conducted at 400–450 µmol m^−2^ s^−1^ of illumination, 75% humidity, and 30°. The experiment was continued for up to 50 days and the sterilized and metal-free water was used based on the requirements.

#### Physiological and biomolecule analyses

During the experimental study, the percentage of germination, biometric properties, and parameters including shoot and root length, width of shoot, and wet and dry biomass (at the end of the experiment) were studied according to Anning and Akoto (2018). Furthermore, the quantity of total proteins, total carbohydrates, and chlorophyll, and the carotenoid content of *C. cajan* (L.) Millsp. from study I, II, and control were studied individually (Nayak et al., 2019). All the parameters were analyzed in triplicate. The SPSS software 16.0 version was used for statistical analysis.

#### Metal analyses of post-treated *C. cajan* (L.) Millsp. biomass

*C. cajan* (L.) Millsp. from study I, II, and control was harvested at the final stage of the study. The harvested plants were rinsed individually with distilled water to wash out the soil and dust particles attached to the surfaces of roots and shoots. Once again, the samples were splashed with 0.1 N HCl for 10 s and subsequently washed with deionized water. The washed plant materials were dehydrated using desiccators and powdered using a pulverizer. The standard acid digestion technique was followed to extract the metal contents present in the roots and shoots of each sample individually. The metals present in the extract were investigated through ICP-OES. The treated and untreated soils were adopted for metal analysis to quantify the volume of the metals reduced in the treated soil by the aforementioned method.

#### Accession number detail of pre-identified *B. cereus* NDRMN001

The nucleic acid sequence of pre-identified *B. cereus* NDRMN001 was submitted to the public database (NCBI) and the accession number for the isolate was obtained as JQ683657.1 (https://blast.ncbi.nlm.nih.gov/Blast.cgi).

## Results

### Physicochemical nature of soil samples

The soil sample collection site is situated 1 km away (downward) from the bauxite mine site. The physicochemical properties of the collected soil sample showed the presence of an enormous amount (mg kg^−1^) of certain heavy metals such as Mn (2,519.6 ± 5.71), Cd (31.24 ± 1.68), Zn (769.57 ± 3.46), Pb (326.85 ± 3.43), and Cr (302.34 ± 1.62) above the permissible limits. The physical factors such as pH (6.8 ± 0.23) and EC (0.4 ± 0.001 dSm^−1^) and K (68.85 ± 3.61) fell within the permissible limits. The other most significant elements, N (96.31 ± 3.28) and P (2.6 ± 0.54), were below the required amount for a healthy plantation (**Table 1**).

**Table 1:** Physicochemical properties of test soil sample compared with control soil.

Soil parameters	Test soil sample	Control soil	Permissible limit
pH	6.8 ± 0.23	7.5 ± 0.74	6–8
EC (dsm^−1^)	0.4 ± 0.001	1 ± 0.03	0.1–1
N mg kg^−1^	96.31 ± 3.28	153.54 ± 2.98	114–180
P	2.6 ± 0.54	8.2 ± 0.53	4.6–9
K	68.85 ± 3.61	98.64 ± 2.41	49–113
Mg	3,652.8 ± 8.43	8,124.8 ± 9.47	52,000
Mn	2,519.6 ± 5.71	864.31 ± 3.94	1,000
Cd	31.24 ± 1.68	5.02 ± 0.39	2–6
Zn	769.57 ± 3.46	462.37 ± 3.18	300–600
Fe	3,594.2 ± 8.61	6,412.8 ± 6.17	129,000
Pb	326.85 ± 3.43	161.35 ± 7.91	200
Cr	302.34 ± 1.62	94.6 ± 1.34	100

The mentioned values are mean and standard error ( ± SE) of triplicates.

### Characterization of PGP traits of B. cereus NDRMN001

PGP traits such as syntheses of NH_3_, HCN, siderophore, and IAA; N fixing; and P solubilization competence of *B. cereus* NDRMN001 were qualitatively and quantitatively analyzed. The attained PGP trait qualitative study results stated that the *B. cereus* NDRMN001 has reasonable PGP traits such as it had the efficiency to produce siderophore, HCN, and IAA. Furthermore, it had the potential to fix N and solubilize P.

### Quantitative analysis of PGP traits

**Figure 2:**
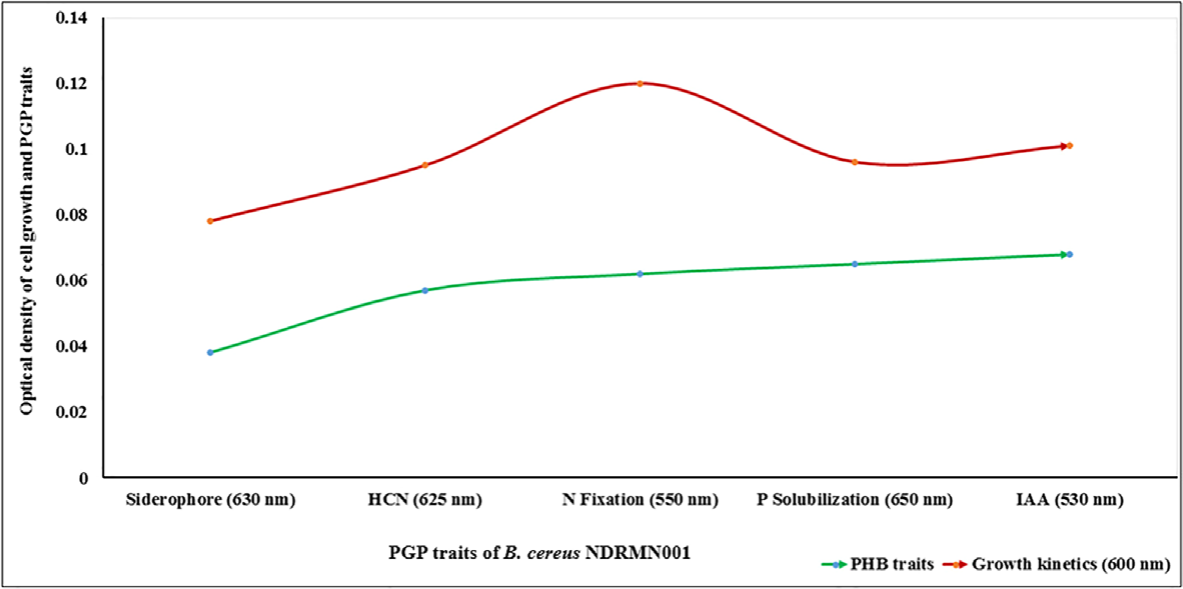
Quantitative analyses of PGP traits of *B. cereus* NDRMN001.

The results attained from the qualitative analysis study stated that the *B. cereus* NDRMN001 had excellent PGP activities except for NH_3_ synthesis. The absorbance-based quantitative analyses were performed to assess the PGP molecules of *B. cereus* NDRMN001 along with bacterial growth. The acquired results stated the significant quantities of PGP molecules such as siderophore (OD: 0.038 at 630 nm) and HCN (OD: 0.057 at 625 nm), N fixation (OD: 0.062), P solubilization (OD: 0.065 at 650 nm), and IAA production (OD: 0.068 at 530 nm) after 4 to 14 days of incubation (**Figure 2**).

### Optimization of growth parameters for PGP traits of B. cereus NDRMN001

**Figure 3:**
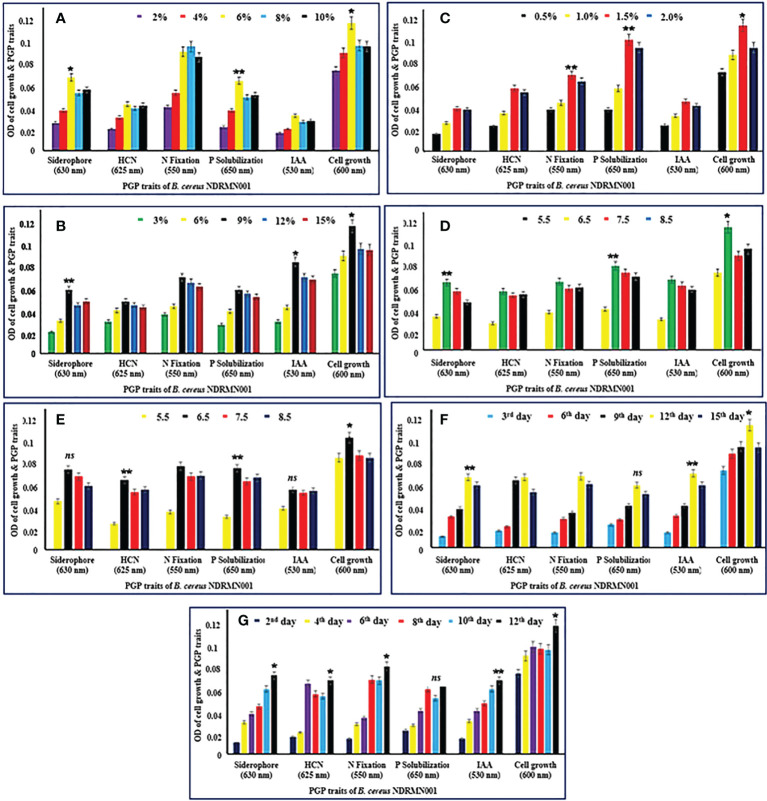
Optimization of growth parameters for PGP activities of *B. cereus* NDRMN001. The mentioned values are mean and standard error ( ± SE) of triplicates. * Significantly differs (*p* < 0.05), ** significantly differs (*p* < 0.001) from others ns, Not significant. **(A)** Various percentage of glucose. **(B)** Various percentage of tryptophan. **(C)** Various percentage of tricalcium phosphate. **(D)** Various pH. **(E)** Various temperature. **(F)** Days of incubation. **(G)** PGP trait production under optimized conditions at different days of interval.

The beneficial traits of bacteria would be produced under optimized growth conditions such as physical (temperature, pH, etc.) and chemical parameters (glucose, tryptophan, etc.). Thus, when bacteria have survived under these appropriate circumstances, their PGP activities would be expressed excellently (**Figures 3A–G**).

### Optimization of the concentration of glucose

The optimized glucose concentration for *B. cereus* NDRMN001 was studied with 2% to 10% of glucose. The attained results stated that the PGP traits of *B. cereus* NDRMN001 were effectively expressed at 6% glucose (**Figure 3A**). At this concentration, the siderophore, HCN, fixed N, soluble P, and IAA had ODs of 0.071, 0.045, 0.096, 0.068, and 0.034, respectively. The significant growth of *B. cereus* NDRMN001 was recorded at 6% of glucose, and it corresponded to the fine PGP trait activities of the bacterium. The acquired data were statistically significant at *p* < 0.01 to *p* < 0.05 compared to the remaining concentrations of glucose. The higher and lower concentrations of glucose might reduce the metabolic activities of *B. cereus* NDRMN001. Hence, the 6% glucose-enriched medium could have enhanced the PGP traits of *B. cereus* NDRMN001.

### Optimization of the concentration of tryptophan

The IAA synthesizing potential of the test isolate was studied with various concentrations (3% to 15%) of tryptophan since tryptophan is highly important and determines the synthesis of IAA in bacteria. Among the various concentrations of tryptophan, the significant growth of *B. cereus* NDRMN001 was observed (OD: 0.124) at 9% tryptophan. Furthermore, with this tremendous growth, PGP traits such as siderophore (OD: 0.062), HCN (OD: 0.051), fixed N (OD: 0.074), soluble P (OD: 0.062), and IAA (OD: 0.089) were statistically significant (at *p* < 0.001 to *p* < 0.05) (**Figure 3B**).

### Optimization of the concentration of tricalcium phosphate

The active PGP traits, especially P solubilization (OD: 0.11) by *B. cereus* NDRMN001, occurred at a 1.5% concentration of tricalcium phosphate. The bacterial growth (OD: 0.124) and other PGP traits such as siderophore (OD: 0.042), HCN (OD: 0.062), fixed N (0.075), and IAA (OD: 0.049) were effective at 1.5% compared to the other concentrations (**Figure 3C**). These were statistically significant at *p* < 0.01 to *p* < 0.05 compared to the other concentrations. This might be related to the biomass of bacteria.

### Suitable pH and temperature

pH as well as temperature are also the most important factors to determine the metabolism, PGP traits, and growth of bacteria (Minari et al., 2020). The suitable pH and temperature for the expression of PGP traits of *B. cereus* NDRMN001 were studied. The obtained results showed that the excellent PGP traits (siderophore: OD 0.068 and 0.062, HCN: OD 0.059 and 0.062, fixed N: OD 0.071 and 0.069, IAA: OD 0.051 and 0.068, and soluble P: OD 0.085) were recorded at pH 6.5 and 30°C (**Figures 3D, E**). These outcomes were statistically significant at *p* < 0.01 to *p* < 0.05 compared to the other pH values and temperatures.

### Suitable incubation time

Among the various growth parameters, the sufficient time duration of incubation had been the most important one to attain the maximum PGP potential of *B. cereus* NDRMN001. Hence, the suitable incubation period for analyzing the PGP traits of the test isolate was evaluated with various incubation periods on 3-day intermittent analysis for 15 days of incubation along with the growth of *B. cereus* NDRMN001. The results stated that most of the PGP traits such as siderophore (OD: 0.071), fixed N (OD: 0.072), soluble P (OD: 0.063), and IAA (OD: 0.075) were influential on the 12th day of incubation and HCN (OD: 0.068) synthesis was effective on the 9th day of incubation (**Figure 3F**). These outcomes were statistically significant at *p* < 0.01 to *p* < 0.05 compared to the others.

### PGP traits under optimized conditions

The extreme PGP traits of *B. cereus* NDRMN001 under the optimized growth conditions (6% glucose, 9% tryptophan, 1.5% tricalcium phosphate, pH 6.5, and temperature 30°) with 12 days of incubation were studied. The obtained PHP traits under these optimized conditions were as follows: siderophore: OD 0.076, HCN: OD 0.071, fixed N: OD 0.085, IAA: OD 0.068, and soluble P: OD 0.071 (**Figure 3G**). These outcomes were statistically significant at *p* < 0.01 to *p* < 0.05 compared to others.

### *B. cereus* NDRMN001 effects on *C. cajan* (L.) Millsp.

**Figure 4:**
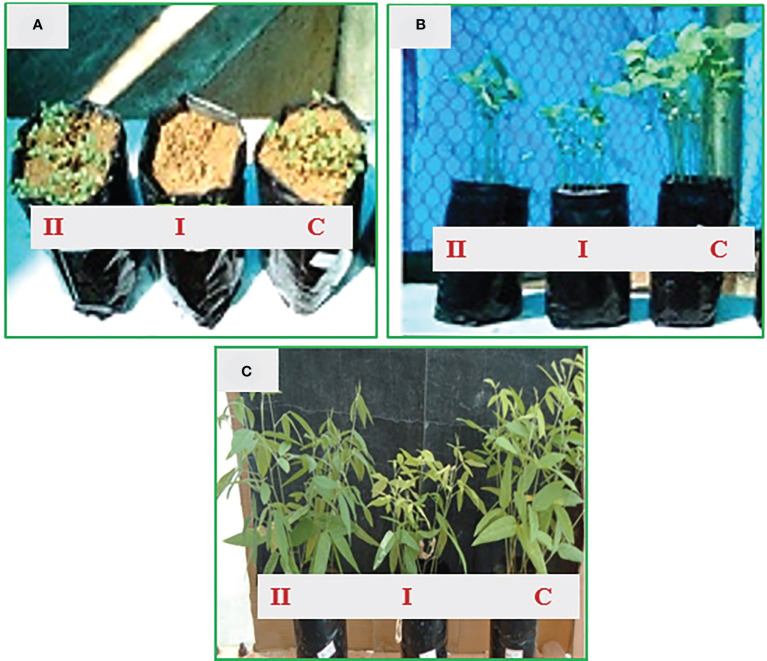
*C. cajan* (*L*.) Millsp. grown under greenhouse experiment with different treatment groups and control. **(A)** 10th day; **(B)** 30th day; **(C)** 50th day.

The optimistic influence of this multi-potential *B. cereus* NDRMN001 on physiological and biomolecule contents of *C. cajan* (L.) Millsp. grown on metal-contaminated soil was studied under greenhouse (**Figure 4**) conditions with the various experimental setups in triplicate.

#### Total chlorophylls and carotenoid

**Figure 5:**
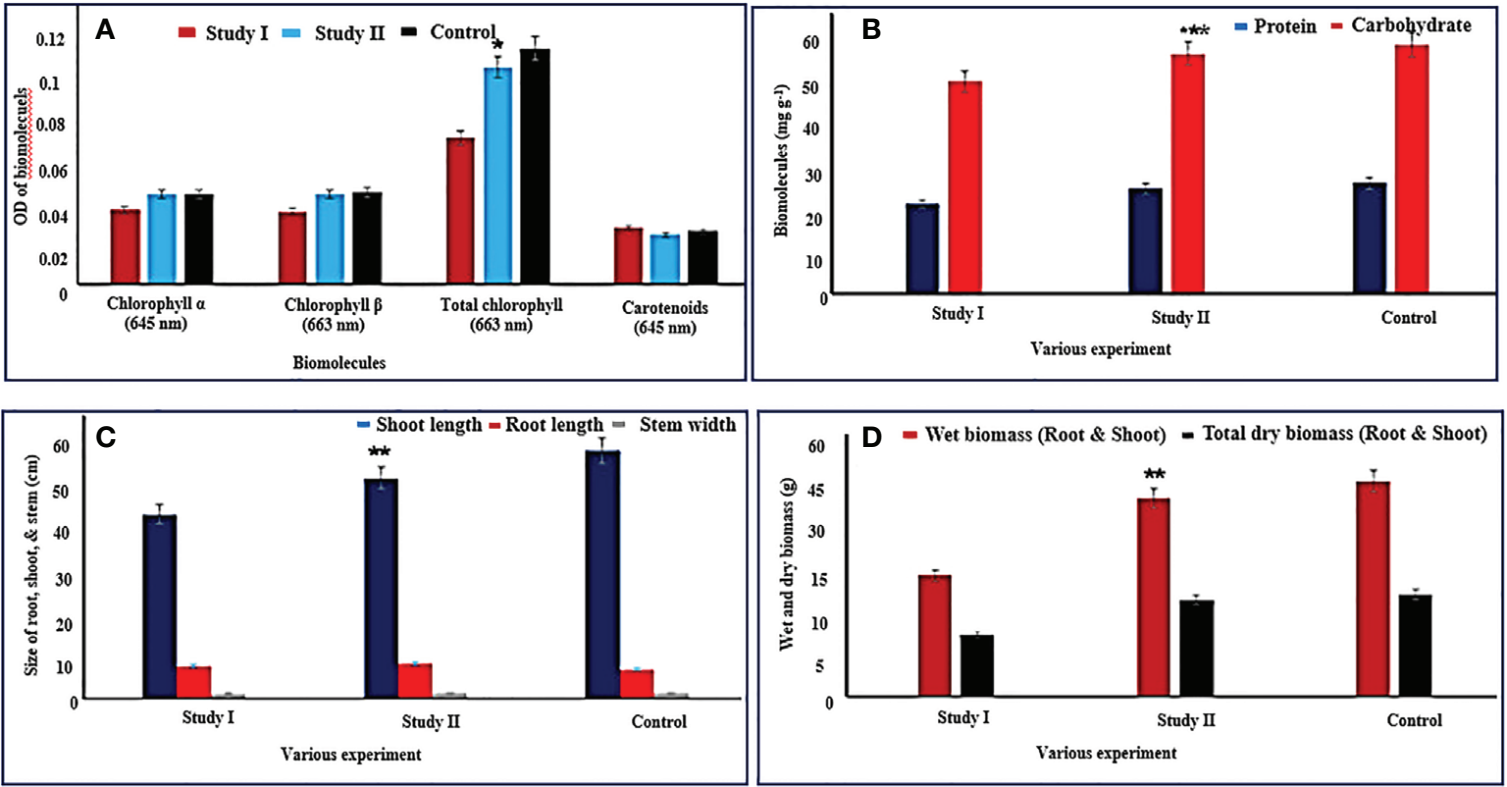
Physiological and biomolecule properties of *C. cajan* (*L*.) Millsp. along with and without the amalgamation of *B. cereus* NDRMN001. The mentioned values are mean and standard error ( ± SE) of triplicates. * Significantly differs (*p* < 0.05), ** and *** significantly differs (*p* < 0.001 and 0.003) from others. **(A, B)** Biomolecule content of *C. cajan* (*L*.) Millsp. from both study I and II compared with control. **(C)** Size of root, shoot, and stem of *C. cajan* (*L*.) Millsp. from both study I and II compared with control. **(D)** Wet and dry biomass of *C. cajan* (*L*.) Millsp. from both study I and II compared with control.

The *C. cajan* (L.) Millsp from experimental study II [*C. cajan* (L.) Millsp. along with *B. cereus* NDRMN001] showed a reasonable quantity of total chlorophyll (OD: 1.24) and carotenoids (OD: 0.36) compared to *C. cajan* (L.) Millsp. (without *B. cereus* NDRMN001) from study I (total chlorophyll: OD: −0.835 and carotenoid: OD: −0.32) (**Figure 5A**). The amount of photosynthetic pigments of *C. cajan* (L.) Millsp. from study II was comparable with the control and statistically significant at *p* < 0.05 compared to study I. This might have been because of the amalgamation of multi-potential *B. cereus* NDRMN001 with *C. cajan* (L.) Millsp. *B. cereus* NDRMN001 reduced the metal toxicity and provided the PGP components to improve the plant growth by maintaining the active biosynthesis of the photosynthetic pigments.

#### Proteins and carbohydrates

Significant amounts (mg g^−1^) of protein (27.18) and carbohydrate (60.13) were noticed in *C. cajan* (L.) Millsp. from study II compared to study I (protein 20.35 and carbohydrate 48.12) and nearly similar to control (**Figure 5B**). Furthermore, these values were significant at *p* < 0.05 compared to study I. This was possible due to the presence of multipotential *B. cereus* NDRMN001 in the rhizosphere region of *C. cajan* (L.) Millsp. that reduced the metal toxicity impact on the plant.

#### Physiological influence

*C. cajan* (L.) Millsp. amalgamated with *B. cereus* NDRMN001 (study II) showed a significant influence on germination (100%), root length (39.13 cm), shoot length (6.23 cm), and width of the stem (1.1 cm) from metal-contaminated soil (**Figure 5C**) and was identical to the control crops grown on fertile soil. The bioinoculant free *C. cajan* (L.) Millsp. from study I showed a reduced length of the shoot (32.74 cm), root (5.85 cm), and width of the stem (1 cm), and it might have been due to the metal stress. The percentage of germination from study I and II was 100%, and it might have been due to the effect of goat manure. The *C. cajan* (L.) Millsp. from *B. cereus* NDRMN001 amalgamated experimental study II showed improved wet (29.84 g) and dry (14.57 g) biomass parallel to the control and greater than study I (bioinoculant free) (wet biomass: 18.23 g and dry biomass 9.37 g) (**Figure 5D**), and these were significant at *p* < 0.05 compared to study I.

#### Phytoremediation potential of C. cajan (L.) Millsp. on metal-polluted soil with the amalgamation of *B. cereus* NDRMN001

In the phytoremediation study, the *C. cajan* (L.) Millsp. from experimental study II had effectively absorbed metals such as Cr (43.17 mg g^−1^), Cd (16.18 mg g^−1^), Zn (47.31 mg g^−1^), Pb (32.23 mg g^−1^), and Mn (23.49 mg g^−1^) from the metal-polluted soil compared to study I (Cr: 7.74, Cd: 12.15, Zn: 16.72, Pb: 11.47, and Mn: 14.52 mg g^−1^) (**Table 2**). Furthermore, the plant growth elements N (142.24), P (4.39), and K (71.28 mg kg^−1^) were increased in study II-treated soil than in study I soil (N: 105.26, P: 2.91, and K: 65.18 mg kg^−1^). The pH of study II soil also showed significant changes (**Table 3**) compared to pre-treated soil. This might be due to the fact that the root of the test plant might synthesize and secrete a considerable volume of secondary metabolites, which may alter the pH of the treated soil. The pre- and post-treated soil samples were also studied, and it was noted that certain amounts of metals had been reduced in the soil and it was significantly interconnected with the amounts of metals absorbed by *C. cajan* (L.) Millsp. The obtained values were significant at *p* < 0.01 to *p* < 0.05 compared to others.

**Table 2:** Phytoremediation competence of *C. juncea* (L.) Millsp.* *in bauxite mine soil with and without the amalgamation of bacterial isolates.

Metals	Pre-treatment (soil)	Experimental study I			Experimental study II		
		Post-treatment	Metal in plant (mg g^−1^)		Post-treatment(Soil)	Metal in plant (mg g^−1^)	
			Root	Shoot		Root	Shoot
Cr	302.34 ± 1.75	294.6 ± 2.67	1.89 ± 0.14	5.85 ± 0.84	259.17* ± 2.67	13.04 ± 0.67	30.13 ± 0.94
Cd	31.24 ± 0.98	19.09 ± 0.35	4.52 ± 0.21	7.63 ± 0.65	15.06* ± 0.61	5.56 ± 0.28	10.62 ± 0.38
Zn	769.57 ± 2.61	752.85 ± 2.63	7.4 ± 0.68	9.32 ± 0.35	722.36** ± 3.95	25.11 ± 0.79	22.10 ± 0.61
Pb	326.85 ± 3.84	315.38 ± 2.96	4.85 ± 0.37	6.62 ± 0.96	294.52** ± 1.98	12.11 ± 0.51	20.12 ± 0.84
Mn	2,519.61 ± 6.95	2,504.58 ± 5.21	12.11 ± 0.69	2.31 ± 0.08	2,349.21** ± 6.58	18.65 ± 0.82	4.84 ± 0.25

The values mentioned in the table were means and ± SE of triplicates. * Significantly dissimilar (p < 0.05), ** significantly varies (p < 0.001) from the pre-treated soil.

**Table 3:** Significant changes in the quantity of essential elements and pH in treated soil compared with control.

Parameters	Study I	Study II	Control
pH	7.9	7.1	7.5
N (mg kg^−1^)	142.24	105.26	295.56
P (mg kg^−1^)	4.39	2.91	2.53
K (mg kg^−1^)	71.28	65.18	195.81

## Discussion

Analysis of the physicochemical parameters revealed that the mine surrounding sites have been severely polluted with heavy metals (**Table 1**). These findings revealed that the distribution of metal pollution from the bauxite mine heaps was geographically positioned in an elevated location relative to the sampled site (Narayanan et al., 2020c) since it is likely to disperse metal pollution heap to surrounding farm sites by weathering activities (Nayak et al., 2019). The farmers are cultivating some crops and simultaneously face less germination and low yield. Hence, the farmers are applying more fertilizers to replace the shortage of essential elements (N and P). This activity might enhance the soil spoilage due to over-dumping of chemical fertilizers (Raza et al., 2020). Moreover, the seedling, which is susceptible to infection, lower yield, etc., could reduce these metal contents’ large volume (Hussain et al., 2019). Besides that, metal pollutants can contaminate the food chain and pass through the uptake of the foods obtained from metal-contaminated sites (Kang et al., 2018). Any crops will extract a few percent of metals in their tissues or people that consume parts of plants (leaf, fruits, etc.) (Guo et al., 2020). This bacterial strain was previously isolated from metal-enriched soil (Narayanan et al., 2020b). Furthermore, the test strain had been reported to have an outstanding metal tolerance, remediation, and solubilization potential on mine soil (Kumar et al., 2019). This characteristic feature of *B. cereus* NDRMN001 could be useful to minimize the metal toxicity in plants and support the germination and growth of plants on metal-polluted soil. The bacteria including *Alcaligenes* sp., *Aeromonas* sp., *Bacillus* sp., and *Pseudomonas* sp. have been reported as HCN producers (Kidd et al., 2017). Another essential PGP trait is IAA production; the existence of the IAA-producing bacterial isolate *B. cereus* NDRMN001 in the rhizosphere region of the plant could enhance the development of roots along with number of root hairs, which facilitate the growth of plants by easy nutrient uptake (Usmani et al., 2019). The N-fixing as well as P-solubilizing potential of bacteria can enhance the availability of the soluble forms of N and P for an easy and comfortable nutrient-absorbing process in plants. Moreover, it would enhance the metabolism process and support plant protection mechanisms (Sharma and Archana, 2016). The siderophore is one of the most essential PGP traits to chelate the iron elements for the growth of plants (Hmaeid et al., 2019). The percentage of siderophore unit (SU) synthesis was derived from the OD value through the SU index as 68.24% SU. The percentage of the SU might vary among the bacterial species; some soil-borne bacteria could produce the siderophore as 9.27% to 62.28% (Devi and Thakur, 2018). The presence of these siderophore-producing isolates in the metal-polluted soil could enhance the plant’s metabolic activity by providing a sufficient quantity of iron molecules, and it could reduce the oxidative stress damage to the plant under metal stress conditions (Das and De, 2018).

The test bacterium had outstanding N-fixing and P-solubilizing potentials; thus, it could aid to increase the germination rate and cell proliferation. The N-fixing native metal-tolerant bacterium and its interactions with the plant are a promising biotechnological approach for the reclamation of degraded land and ecosystems (Ferreira et al., 2012). Similarly, few studies have previously reported that the bacteria isolated from the mining had metal tolerance as well as PGP activities (Ferreira et al., 2012). This *B. cereus* NDRMN001 fixed the N in a soluble state; thus, the plant could easily utilize the soluble form of N and provide secondary metabolites from the root region; eco-friendly phytoremediation is more feasible by this interaction of plant bacteria with metal exposure sites (Enebe and Babalola, 2018). Moreover, P is the most essential factor for a healthy plantation (Dogra et al., 2019). The test isolate had the superior P-solubilizing potential; it mobilized the insoluble P (rock P) into soluble P and made it easy for plant utilization (Asemoloye et al., 2017). The most familiar bacterial species recorded to date to be potent P solubilizers are *Acinetobacter* sp., *Bacillus* sp., *Burkholderia* sp., *Alcaligenes* sp., *Flavobacterium* sp., etc. (Das and De, 2018). In most cases, the naturally available rock P and superphosphate are associated with calcium, and when the pH of the soil fluctuates, other geochemical conversions lead to P precipitation in the form of CaHPO_4_ or CaHPO_4_ 2H_2_O (Fahad et al., 2015). Thus, the availability of metal-tolerant and P-solubilizing bacteria with the plant seedling in a metal-polluted site could be useful for the phytoremediation process (Li et al., 2019). The IAA synthesized by *B. cereus* NDRMN001 by utilizing 3% tryptophan corresponded to 45.21 µg ml^−1^. Similarly, Devi and Thakur (2018) reported that 21 isolates enumerated from the soil sample possessed IAA, producing efficiency up to 1.6–47.56 μg ml^−1^ with the consumption of 5 mg ml^−1^ of tryptophan. The availability of IAA-producing bacteria in the stressed environment could provide moderate growth through the significant proliferation of cell division, especially in the root region; it supports the lengthening of roots and development of root hairs (Alka et al., 2020). It supports the gradual absorption of water and nutrients from the metal-polluted sites (Enebe and Babalola, 2018). Carbon sources such as glucose are a major nutritional factor that determines the metabolism and growth of bacteria (Bruno et al., 2020). Nevertheless, the quantity of glucose in a medium is also the most significant factor to promote the PGP traits of the bacterium (Das and De, 2018). Hence, a 6% glucose-enriched medium could have enhanced the PGP traits of *B. cereus* NDRMN001. It might be because glucose is a ready-made and energy-enriched carbon source, and the bacterium could have utilized the glucose gradually and maintained its metabolism in a steady-state manner (Dogra et al., 2019). This indicated that the *B. cereus* NDRMN001 might have a tryptophan-dependent IAA pathway (Etesami, 2018). This test bacterium might have possessed tryptophan-metabolizing enzymes such as tryptophan-2-monooxygenase and IAM-hydrolase (Alka et al., 2020). Initially, the tryptophan might have been reduced to indole-3-acetamide (IAM) through tryptophan-2-monooxygenase and the final IAA might have been derived from IAM by IAM-hydrolase (Garg and Aggarwal, 2011). Hence, this 9% tryptophan concentration effectively facilitated the IAA synthesis in *B. cereus* NDRMN001. This IAA could have acted as active auxins to support active root growth and root hair proliferation and plant growth (Gong et al., 2018).

Phosphate is one of the essential factors for the active growth of plants and biological activities in soil (Asad et al., 2019). The soluble form of P could support plant metabolism and develop resistance in plants against drought, flood, disease, etc. (Sharma and Archana, 2016). Hence, the availability of soluble P is more important (Sharaff et al., 2017). The P-solubilizing feature of *B. cereus* NDRMN001 could be useful to improve the soil ecosystem and fertility, and support plant growth (Li et al., 2019). Numerous bacterial species such as *Azotobacter* sp., *Azospirillum* sp., *Bacillus* sp., and *Burkholderia* sp. have been identified as potential P-solubilizing rhizobacteria that could enhance soil fertility and plant growth (Ma et al., 2013). pH and temperature are also the most significant factors to determine the metabolism, PGP traits, and growth of bacteria (Minari et al., 2020). A suitable pH is a major important factor to improve the PGP traits of *B. cereus* NDRMN001 since it regulates the bioavailability of basic essential nutritional contents and enhances the activities of the enzymes (Ma et al., 2015). Each enzyme-related metabolic activity is directly related to the pH conditions of the bacterial surroundings (Hmaeid et al., 2019). The pH requirement of bacteria might vary from acidic to alkaline, based on the origin (Kang et al., 2018). Similarly, the *B. cereus* NDRMN001 was isolated from slightly acidic environmental conditions like pH 6. Correspondingly, the effective PGP traits were expressed at pH 6.5. The optimized temperature could activate and maintain internal cell organelle activities and chelate certain metabolic activities and cell proliferation (Li et al., 2019). The temperature changes among the bacteria might be derived from their inheritance, and temperature determines the enzymatic activities involved in the PGP traits of *B. cereus* NDRMN001 (Ma et al., 2015). Hence, pH and temperature are the major factors of concern for the effective PGP traits of *B. cereus* NDRMN001. The growth of *B. cereus* NDRMN001 was also noticed on the 12th day of incubation. Such results suggested that the maximum PGP potential of *B. cereus* NDRMN001 was expressed after 12 days of incubation. The maximum PGP traits corresponded to the biomass of *B. cereus* NDRMN001 (Narayanan et al., 2020b).

These entire results confirmed that the *B. cereus* NDRMN001 could serve as the most suitable PGP bacterium (Hmaeid et al., 2019). It was previously identified that it had metal tolerance and degradation potential and could be applied on metal-polluted soils as a bioremediation agent and soil fertility-enhancing PGP bacterium (Narayanan et al., 2020c). Therefore, this multi-potential *B. cereus* NDRMN001 could support and improve the phytoremediation potential of the plant by reducing the metal toxicity and providing the PGP components under metal stress conditions (Kang et al., 2018). The chlorophylls (including *a* and *b*) and carotenoids are pivotal photosynthetic pigments in plants used to convert solar energy to chemical energy through the photosynthesis process (Raza et al., 2020). In most plants, the photosynthetic pigment secretion process is susceptible to metal stress (Hmaeid et al., 2019). The earliest impact of metals on plants is damaging the photosynthetic pigments by causing chlorosis and retardation (Asemoloye et al., 2017). *B. cereus* NDRMN001 reduced the metal toxicity and provided the PGP components to improve plant growth by maintaining the active biosynthesis of the photosynthetic pigments (Hussain et al., 2019; Allamin et al., 2020).

### Possible biochemical reactions

The metal-tolerant and degradation/solubilization properties of *B. cereus* NDRMN001 blended with *C. cajan* (L.) Millsp. can reduce metal toxicity, and their PGP traits delay the senescence process of the plant through the accumulation of potassium ions, the reduction of ethylene synthesis, and the induction of growth hormones such as IAA, which prevent senescence, enhance the accretion of carbohydrates and proteins, and support the plant survival under metal stress conditions (Enebe and Babalola, 2018). Furthermore, it facilitates a rapid recovery from metal stress than the plant without bioinoculants (Mathiyazhagan and Natarajan, 2013). This positive relationship contributes to the accumulation of carbohydrates in the plant and, during stress, reserves osmolytes and helps the plant stay green (Jerez Ch and Romero, 2016; Kang et al., 2018). Similarly, *Arachis hypogaea* amalgamated with *Bradyrhizobium* sp. under abiotic stress conditions had huge amounts of proteins and carbohydrates achieved by the nitrogenase catalyzed (N fixation) process in the rhizosphere region leading to the accumulation of proteins and carbohydrates in the plant (Pandey et al., 2018). The metal tolerance and active PGP traits of *B. cereus* NDRMN001 enhanced the growth of the plant by improving the root and shoot lengths *via* IAA synthesis (Pinto et al., 2018) and by offering sufficient and soluble N and P molecules through N fixation and P solubilization (Prakash et al., 2019). Similarly, *P. fluorescens* amalgamated cowpea plant had lengthy roots and shoots compared with the non-bioinoculant cowpea plant in an abiotic stress environment (Manaf and Zayed, 2015). These properties improved the photosynthetic pigments and photosynthesis and led to more biomass of *C. cajan* (L.) Millsp. (Garg and Aggarwal, 2011). The obtained results correlated with the findings of Manaf and Zayed (2015). They reported that the endomycorrhizae and *P. fluorescens* inoculated with *Vigna unguiculata* under a metal stressed environment showed higher wet as well as dry biomass.

The addition of the multi-potential *B. cereus* NDRMN001 considerably improved the phytoremediation competence of *C. cajan* (L.) Millsp. This was possible due to the metal tolerance and PGP traits (enhanced the plant metabolic activities) of *B. cereus* NDRMN001 (Checcucci et al., 2017). Similarly, another study reported the stimulus effect of rhizobacteria in phytoremediation efficiency of *Helianthus* on metal-contaminated soil (Kong and Glick, 2017).

## Conclusion

The pre-isolated multi-metal-tolerant *B. cereus* NDRMN001 possesses remarkable PGP traits such as secretion of HCN, siderophore, IAA, N fixing, and P solubilization. The optimum growth parameters required for the expression of PGP traits were optimized. The *C. cajan* (L.) Millsp. inoculated with *B. cereus* NDRMN001 (study II) showed excellent physiology and biomolecule contents and effectively absorbed the metals from the soil and accumulated them in the root and shoot regions compared to the plant without the bacterial inoculum (study I). These outcomes concluded that this optimistic interaction among *C. cajan* (L.) Millsp. and *B. cereus* NDRMN001 could be an effective sustainable approach to removing the metal pollutants from the contaminated site.

## Data availability statement

The datasets presented in this study can be found in online repositories. The names of the repository/repositories and accession number(s) can be found below: https://www.ncbi.nlm.nih.gov/, JQ683657.1.

## Author contributions

All authors contributed to the article and approved the submitted version.

## Funding

The authors extend their appreciation to the Deanship of Scientific Research, Ton Duc Thang University, Ho Chi Minh City, Vietnam. This work is carried out at the College of Resources and Environment, Southwest University, supported by the Fundamental Research Funds for the Central Universities of China (No. SWU 020010), the Natural Science Foundation of Chongqing (No. cstc2021jcyj-msxmX0827), the Returned Overseas Students Entrepreneurship and Innovation Support Program of Chongqing, China (No. cx2021001) and the Science and Technology Research Program of Chongqing Municipal Education Commission, China (KJZD-K202200204).

## Conflict of interest

The authors declare that the research was conducted in the absence of any commercial or financial relationships that could be construed as a potential conflict of interest.

## Publisher’s note

All claims expressed in this article are solely those of the authors and do not necessarily represent those of their affiliated organizations, or those of the publisher, the editors and the reviewers. Any product that may be evaluated in this article, or claim that may be made by its manufacturer, is not guaranteed or endorsed by the publisher.
